# Too Many Shades of Grey: Photometrically and Spectrally Mismatched Targets and Backgrounds in Printed Acuity Tests for Infants and Young Children

**DOI:** 10.1167/tvst.9.12.12

**Published:** 2020-11-06

**Authors:** Guillermo Vivas-Mateos, Iain A. T. Livingstone, Ruth Hamilton, Arsalan Cheema, Mario E. Giardini

**Affiliations:** 1 Department of Biomedical Engineering, University of Strathclyde, Glasgow, Scotland, UK; 2 NHS Forth Valley, Falkirk Community Hospital, Falkirk, Scotland, UK; 3 Royal Hospital for Children, NHS Greater Glasgow & Clyde, Glasgow, Scotland, UK; 4 College of Medical, Veterinary & Life Sciences, University of Glasgow, Glasgow, Scotland, UK

**Keywords:** visual acuity, Teller Cards, Keeler Acuity Cards, Lea Paddles, Cardiff Cards, Peekaboo Vision

## Abstract

**Purpose:**

Acuity tests for infants and young children use preferential looking methods that require a perceptual match of brightness and color between grey background and target spatial average. As a first step in exploring this matching, this article measures photometric and colorimetric matches in these acuity tests.

**Methods:**

The luminance, uniformity, contrast, and color spectra of Teller Acuity Cards, Keeler Acuity Cards for Infants, and Lea Paddles under ambient, warm, and cold lighting, and of grey-emulating patterns on four digital displays, were measured. Five normal adults’ acuities were tested at 10 m observationally.

**Results:**

Luminance and spectral mismatches between target and background were found for the printed tests (Weber contrasts of 0.3% [Teller Acuity Cards], −1.7% [Keeler Acuity Cards for Infants], and −26% [Lea Paddles]). Lighting condition had little effect on contrast, and all printed tests and digital displays met established adult test luminance and uniformity standards. Digital display grey backgrounds had very similar luminance and color whether generated by a checkerboard, vertical grating, or horizontal grating. Improbably good psychophysical acuities (better than −0.300 logMAR: (logarithm of the minimum angle of resolution)) were recorded from adults using the printed tests at 10 m, but not using the digital test Peekaboo Vision.

**Conclusions:**

Perceptible contrast between target and background could lead to an incorrectly measured, excessively good acuity. It is not clear whether the luminance and spectral contrasts described here have clinically meaningful consequences for the target patient group, but they may be avoidable using digital tests.

**Translational Relevance:**

Current clinical infant acuity tests present photometric mismatches that may return inaccurate testing results.

## Introduction

**Figure 1.**
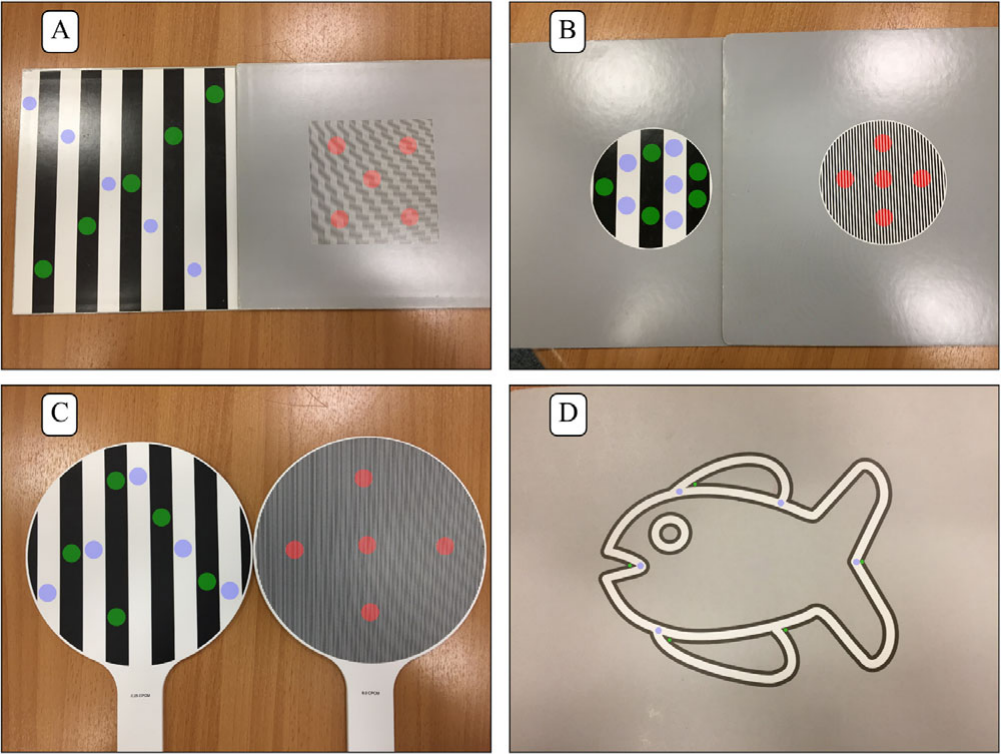
Location of the measurement points for the printed tests. Red dots: grating zones of high spatial frequency targets (at 25% or 50% of the target diameter or diagonal); blue and green dots: white and black zones, respectively, of low spatial frequency targets, chosen to be distributed evenly across the target area. (A) Teller Acuity Card (TAC), (B) Keeler Acuity Cards for Infant (KACI), (C) Lea Paddles (LP), and (D) Cardiff Acuity Cards (CAC).

Acuity testing of infants and children is widely used for diagnostic and screening purposes. Most acuity tests for infants and young children use preferential looking methods with high contrast targets on an isoluminant grey background: if the target is perceptible, infants tend to give behavioral cues such as looking toward the target preferentially. This method underpins printed tests (Teller Acuity Cards, first commercial version [TAC - Vistech Consultants Inc. Ohio, USA, batch no. 1277], Keeler Acuity Cards for Infants [KACI - Keeler, Windsor, UK], Lea Paddles [LP - Lea-Test Ltd and Good-Lite, Elgin, IL]), and a tablet-based digital acuity test, Peekaboo Vision (PV - Scottish Health Innovations Ltd, Clydebank, UK)^1^^,^^2^ (Figs. 1A–C). Cardiff Acuity Cards (CAC - Good-Lite) use a vanishing optotype method^3^ where the target object's outline (car, duck, boat, apple, or house) is a white line bordered by darker lines half as thick (Fig. 1D) whose size is used to infer a mean angle of resolution, typically expressed in Snellen equivalent.

For these tests, the grey background must be an exact perceptual brightness and color match of the spatial average luminance of the high contrast target. The target area is typically much larger than the spatial frequencies of its grating or image. Any luminance mismatch between the background and the target creates a contrast difference that, if perceptible, could trigger an invalid looking response and produce an artificially good acuity result.

Although targets and backgrounds are nominally black, white or grey, any difference in dominance of a particular color will create a color contrast between target and background, and, if perceptible, could similarly trigger a looking response and an artificially good acuity result. Such color properties are affected by the spectral characteristics of the ambient illumination, which can vary significantly within test settings, for example, fluorescent clinic lighting, LEDs, incandescent bulbs, daylight, or some combination thereof.

Photometry and colorimetry are objective and repeatable means of quantifying luminance and color. It may be that minor luminance or color mismatches are not perceived by the target clinical group and, therefore, have negligible clinical impact. However, an initial step in understanding any potential for false-positive looking responses in infant acuity tests is to undertake photometric and colorimetric measurements, and these measurements are the aim of the present study. We aim to evaluate acuity tests for infants and young children in terms of the luminance and spectral composition of their composite elements (black, white, and grey) and to test the assumption of background grey matching spatial average luminance, and color of targets. We used three illumination conditions: ambient fluorescent light, similar to a typical clinical setting, and cold (daylight) and warm (incandescent) light. Although national and international standards for luminance and luminance uniformity exist for adult acuity tests,^4^^,^^5^ no standards exist for acuity tests for infants and young children. We evaluated the extent to which these acuity tests meet adult acuity test standards. We evaluated three high-resolution black and white patterns to generate the pseudo-grey background on the digital screen acuity test: checkerboard, vertical, or horizontal bar gratings at maximum device resolution.

We undertook a limited, brief observational assessment by measuring visual acuity from five healthy, normally-sighted adults using acuity tests at a large distance (10 m) under all three lighting conditions.

## Methods

### Physical Properties of the Cards and Screens

Luminance and spectral properties of the target patterns and backgrounds were measured for four printed tests and four digital displays. The four printed tests assessed were the Teller Acuity Cards (TAC), Keeler Acuity Cards for Infants (KACI), Lea Paddles (LP), and Cardiff Acuity Cards (CAC) (Fig. 1). The digital displays assessed were: iPhone 6 and iPad 3 (Apple Inc, Cupertino, CA); a laptop screen (MSI GL62M 7RD; Micro-Star Int'l Co. Ltd., New Taipei, Taiwan), and a 4K HD monitor (Philips BDM4350; Koninklijke Philips N.V., Amsterdam, the Netherlands).

**Figure 2.**
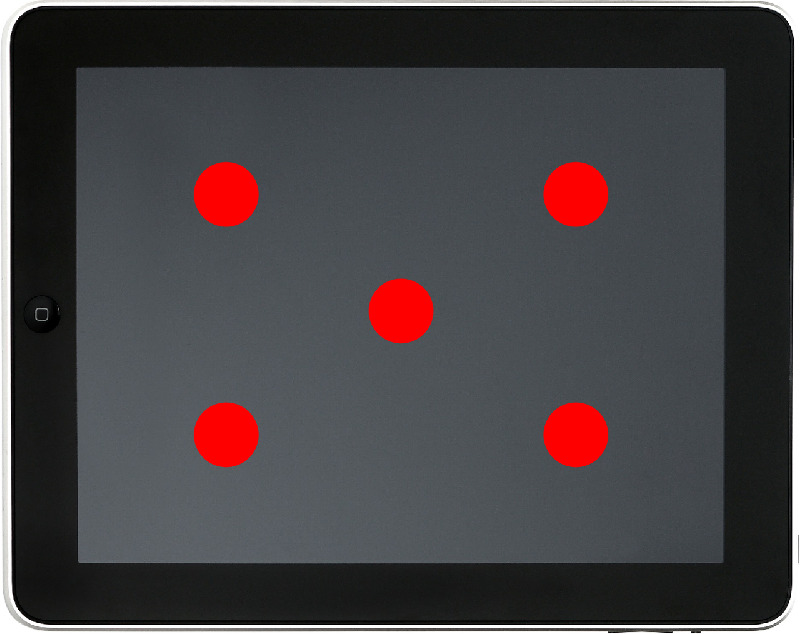
Location of the measurement points for the digital displays. Each point is located at a distance of 25% of the two nearest screen edges, except for the middle point, which is located at 50% of distance of all the screen edges.

Five locations were measured across the digital displays (Fig. 2) that were turned on for at least 15 minutes before measurements^6^ and set to a screen brightness of 50% with auto-brightness turned off, which has previously been shown to meet the International Council of Ophthalmology Early Treatment Diabetic Retinopathy Study mean luminance standards.^1^ Measurement points were located a set proportion away from the screen edges in each case to account for different screen sizes. Because the printed tests had different target shapes owing to the nature of the tests, measurement locations differed by test (Fig. 1).

#### Luminance

**Figure 3.**
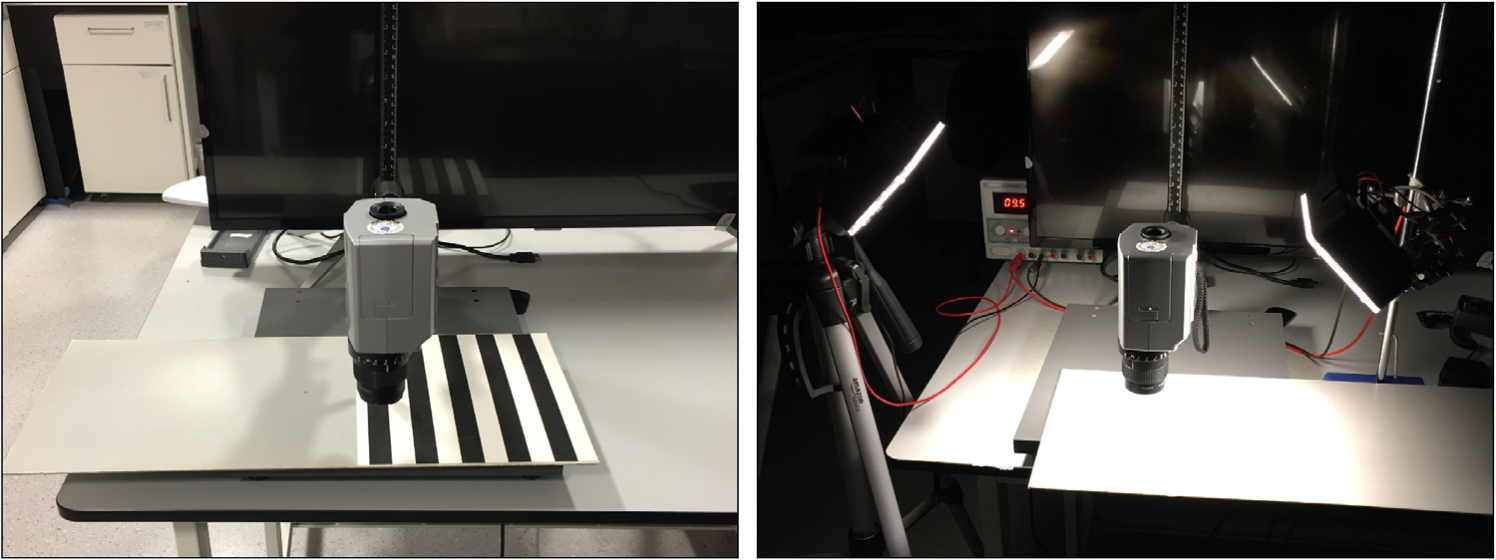
Layout for the luminance measurements under ambient light (*left*) and under studio lights (*right*).

Luminance measurements were performed with a luminance meter (Minolta LS-100, Konica Minolta Sensing, Europe B.V.) with a 1° aperture, close-up lens (No. 110) and calibration traceable to the Japanese national primary standard. The targets were placed on a horizontal surface with the luminance meter lens perpendicularly above at a distance of 71 ± 5 mm, with attention to minimize any cast shadows (Fig. 3). Three illumination conditions were created. Ambient light (ambient) used fluorescent bulbs (Sylvania CF-LE 40W, LEDVANCE, Wilmington, MA) to represent a typical clinic room (Fig. 3, left). Two LED studio lights with tunable color temperature and high color rendition index (Aputure Amaran AL-H198C; Aputure, Shenzhen, China) were positioned 45° to the target surface, illuminating the target from two sides (Fig. 3, right) and generating cold light (cold) at 5500K, similar to daylight, and warm light (warm) at 3200K, similar to incandescent bulbs. Natural daylight was excluded completely.

Illuminance was measured with a light meter (ISO-TECH ILM-01; RS Components, Corby, UK) at 0, 15, and 30 minutes after starting the luminance readings. Studio lights were switched on 30 minutes before the measurements to allow stabilization. Average illuminance under ambient, cold, and warm conditions were 450, 3682, and 3432 lux, respectively, with variations of approximately 1% or less of the average value over the course of the measurements.

**Figure 4.**
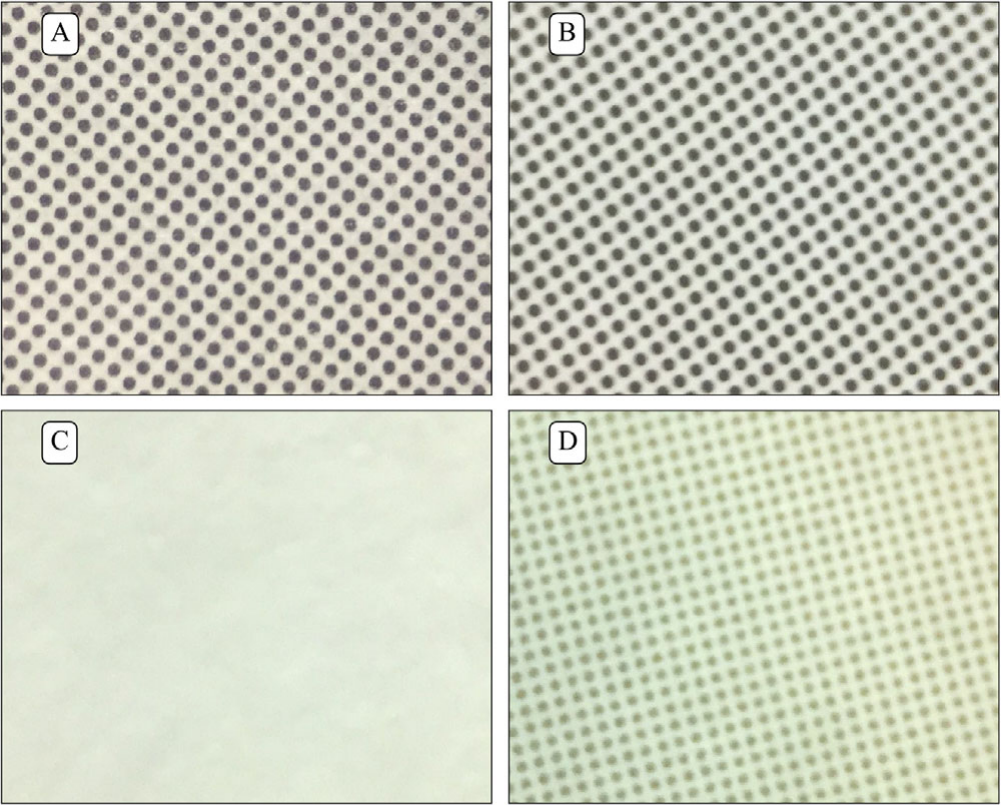
Close-up of the grey background for the four printed tests. (A) Teller Acuity Card (TAC), (B) Keeler Acuity Cards for Infant (KACI), (C) Lea Paddles (LP), and (D) Cardiff Acuity Cards (CAC). Backgrounds are black dots printed over a white background for all tests except the LP.

For the four printed tests, five luminance measures were made under each of the three lighting conditions: white of coarsest grating, black of coarsest grating, grey background, average (over field of view) of second finest grating, and average (over field of view) of finest grating. There were two exceptions to this: (1) It was not possible to measure average (over field of view) of second finest or finest gratings for the CAC test owing to the small size of the target details, i.e. three fine lines rather than an extended grating. (2) It was not possible to measure average (over field of view) of second finest grating of the LP test with the close-up lens because the spatial frequency was too low for consistent measurement. It could be measured without the close-up lens, at 106 ± 1 cm, normalizing subsequently to the luminance of the close-up lens condition. Three printed tests (TAC, KACI, and CAC) did not have homogenous grey backgrounds, but were printed as a pattern of black dots over a white background (Fig. 4).

**Figure 5.**
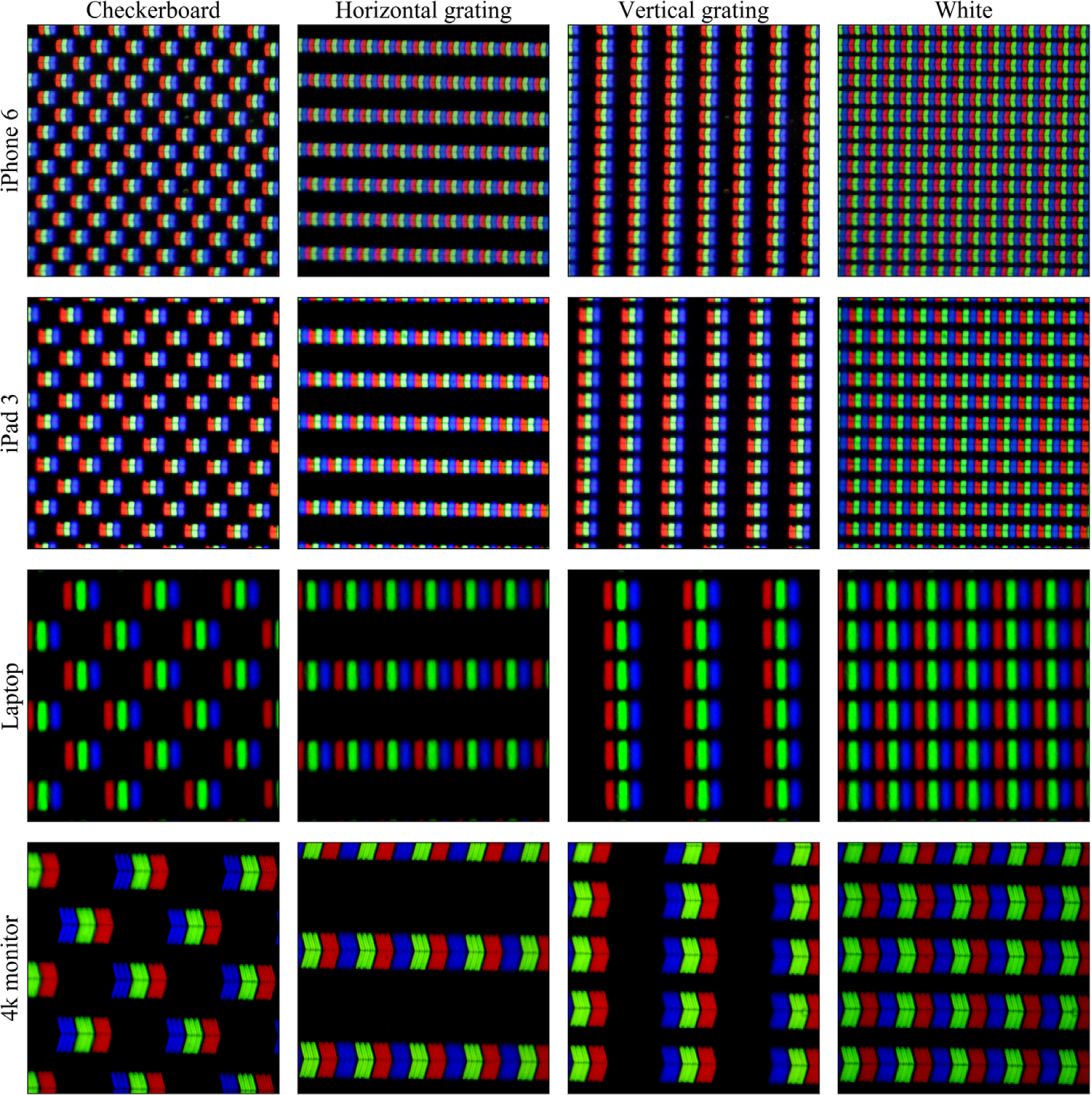
Close-up of the pixels of the digital displays. The rows represent, in order (*upper to lower*): iPhone 6, iPad 3, laptop, and 4K monitor. The columns represent, in order (*left to right*): checkerboard, horizontal grating, vertical grating, and white.

For the four digital displays, five luminance measures were made under two lighting conditions (ambient lights on and off), but not under warm or cold conditions because ambient illumination does not affect emission spectra. The five measures were white of full white screen, black of full black screen, and three greys: a checkerboard pattern, a vertical grating, and a horizontal grating, each generated using the OpenCV computer vision libraries (https://opencv.org/) at the maximum resolution of each device, that is, each half-cycle of the grating was 1 pixel, combining white (RGB 255, 255, 255) and black (RGB 0, 0, 0) pixels (Fig. 5). These fine grating patterns have spatial frequencies below visual acuity limit^7^ at an appropriate distance. For reference, when the screens display the finest possible grating (1 pixel wide alternating black and white lines/checkers; i.e., two-pixel wide line pairs), a subtended angular frequency of 60 cy/deg is obtained at 536 mm, 661 mm, 1236 mm, and 1704 mm for the iPhone 6, iPad 3, laptop, and 4K monitor displays, respectively.

For the printed tests, Weber contrast was calculated from luminance of grey background (*L_background_*) and gratings (*L_grating_*) as

(1) $$\frac{L_{grating}-\;L_{background}}{L_{background}}$$

and expressed as a percentage.

Digital devices can simulate a grey background using the same grating/checkerboard patterns that were measured in this study.^7^ Because of this, calculation of their contrast (Equation 1) was considered redundant.

Uniformity was calculated from the maximum (*L_white__max_*) and minimum luminance of white (*L_white__min_*) for each test and lighting condition as

(2) $$\frac{L_{white\;min}}{L_{white\;max}}$$

Compliance of overall luminance with International Council of Ophthalmology luminance standard^4^ (>80 cd/m^2^), with BS 4274–1:2003 standard^5^ (>120 cd/m^2^) and of luminous uniformity with BS 4274–1:2003 standard^5^ (>80%) for acuity tests was assessed.

#### Spectrometry

Spectral measurements were made using a compact spectrometer (FLAME-S-XR1, Ocean Optics, Largo, Florida), with a range of 200 to 1025 nm, with an optic fiber cable (QP600-1-VIS-NIR, Ocean Optics) of 600 µm core diameter. Spectral measurements of three of the printed tests (TAC, KACI, and LP) were done under the same three lighting conditions used for the luminance measurements (ambient, cold, and warm), but illuminance of the ambient light was slightly lower (347 vs. 450 lux). Spectral measurements of the CAC were not made for the same reasons given in the Luminance section.

Normalized differential reflected light spectra for the three printed tests were calculated from the spectral data as the difference between the normalized spectrum of light reflected by the grey background and by the finest grating target area. This process aimed to highlight any spectral differences between the background and the target that might underpin a perceived color difference. Similarly, normalized differential emitted light spectra were calculated for all of the grey-emulating digital patterns and compared among themselves (checkerboard vs. horizontal, checkerboard vs. vertical, vertical vs. horizontal).

### Psychophysical Tests

Five young (range, 22–28 years) adult subjects, two male, were recruited from staff and students and gave prior written consent. None of the authors took part as a study participant. The experiment was approved by the University of Strathclyde Research Ethics Committee in accordance with the Declaration of Helsinki (application number DEC/BioMed/2019/267). Subjects were screened for normal visual acuity (6/6 or better, iSight Test Pro crowded Early Treatment Diabetic Retinopathy Study) and normal color vision (at least 11 of 12 digital Ishihara plates). Each subject performed all five acuity tests (TAC, KACI, LP, CAC, and PV [iPad 3 only]) with test order pseudorandomized and equally balanced, repeated under three lighting conditions. Subjects were tested with both eyes open, wearing habitual refractive correction as needed.

To capture any exceptional anomalous acuity results, the test distance was 10 m, using a plane viewing mirror owing to the dimensions of the test room. This test distance is at least 10 times greater than the furthest recommended distance of any of the tests considered in the present work. Detection thresholds for each test were determined using a descending method of limits (coarse to fine), with lower spatial frequency targets presented only once. When subjects first incorrectly identified a target, that card and subsequent higher spatial frequency cards were each presented ten times until a subject identified 5 or fewer of the 10 correctly (chance level performance). Testers instructed subjects to indicate “on which side do you see the pattern?” and mandated a “best guess” if the subject did not know, and did not disclose whether choices were correct. A time limit of 10 s per card or level was used to keep overall test times manageable and subjects were encouraged to take breaks if needed. Threshold was defined as 8 correct results out of 10 presentations (binomial probability of 0.04).

For the CAC test, correctly naming the object was assumed to mean a subject could see that level, and the next level was tested. If the target could not be correctly named, a forced choice method was used, with subjects indicating whether the target was at the top or bottom of the card.

For the PV test, a two-target rather than four-target, setting^2^^,^^7^ was selected to match the printed tests and to limit confounding influence relating to the number of possible positions of the target.

The angle subtended at the eye by the threshold grating or vanishing optotype composite line was calculated (minimum angle of resolution) and expressed as a logarithm of the minimum angle of resolution (logMAR) score.

## Results

### Luminance

Black gratings showed the greatest relative difference across the printed tests, and white gratings showed the lowest relative difference; this finding was consistent for all lighting conditions. Luminance of grey backgrounds and finest or second finest gratings did not always match closely, especially for the LP test. As expected, lighting condition affected luminance of all tests and measured areas, with the relatively dim ambient fluorescent lighting resulting in much lower luminances than the brighter cold or warm lighting (Table 1).

**Table 1.** Average Luminance (cd/m^2^) of Black of Coarsest Grating, White of Coarsest Grating, Grey Background, Finest Grating, and Second Finest Grating

	Ambient Light (450 lux)					Cold Light (5500K, 3682 lux)					Warm Light (3200K, 3432 lux)				
	Black	White	Grey	Finest Grating	Second Finest Grating	Black	White	Grey	Finest Grating	Second Finest Grating	Black	White	Grey	Finest Grating	Second Finest Grating
TAC	7.5	129	59–72^a^	68	56	56	1038	472–571^a^	542	450	52	950	431–519^a^	496	412
KACI	3.1	128	59–60^a^	59	59	25	1028	478–483^a^	476	482	22	934	435–449^a^	434	420
LP	7.4	136	66	49	58^b^	52	1079	532	388	468^b^	48	1002	480	352	430^b^
CAC	21	125	69	NM	NM	162	984	545	NM	NM	155	906	501	NM	NM

TAC: Teller Acuity Cards, KACI: Keeler Acuity Cards for Infants, LP: Lea Paddles, CAC: Cardiff Acuity Cards, NM, not measurable; see Luminance.

a Luminance of grey backgrounds varied (see Table 2).

b Measured without close-up lens, normalized to close-up lens condition; see Luminance.

The luminance of the grey backgrounds differed by card for the TAC test, noted to be part of the manufacturing quality control process,^8^ but much less so for the KACI test (Table 2).

**Table 2.** Average Luminance (cd/m^2^) of the Grey Backgrounds on Three Different Cards of the Teller Acuity Card (TAC) Test and the Keeler Acuity Cards for Infant (KACI) Test

	Ambient Light (450 Lux)			Cold Light (5500K, 3682 Lux)			Warm Light (3200K, 3432 Lux)		
	Coarsest Grating Card	Finest Grating Card	Second Finest Grating Card	Coarsest Grating Card	Finest Grating Card	Second Finest Grating Card	Coarsest Grating Card	Finest Grating Card	Second Finest Grating Card
TAC	72	67	59	571	528	472	519	487	431
KACI	60	60	59	482	483	478	439	449	435

**Figure 6.**
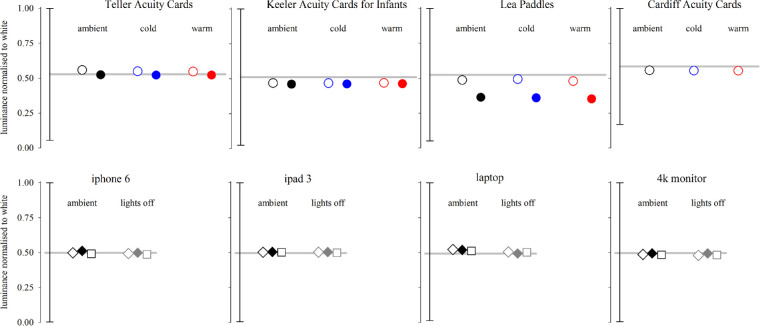
Relative luminance (normalized to white) for the four printed tests (*upper panels*) and the four digital displays (*lower panels*). The relative luminance of black and white for each test and display is shown as the vertical bar on the left of each panel and the theoretical luminance of the ideal grey of the grey background is shown as a grey horizontal line in each panel. Measured, normalized luminances are shown as symbols. *Upper panels*: open circles - grey background; closed circles - finest grating. *Lower panels* show luminance of grey backgrounds generated by different patterns: open diamond - horizontal grating; closed diamonds - vertical grating; squares - checkerboard grating.

For the digital displays, turning the ambient, fluorescent lights off did not noticeably change their luminance other than to decrease the luminance of black areas a little, as might be expected for emissive rather than reflective surfaces. Greys generated by all three grating patterns had similar luminances within each display (Table 3, Fig. 6 lower panels) and closely matched the theoretical luminance of the ideal grey of the grey background based on each display's black and white values.

**Table 3.** Average Luminance (cd/m^2^) for the Digital Displays of Pure Black, Pure White, and Three Grey Backgrounds

	Ambient Light On (450 Lux)					Ambient Light Off				
	Black	White	Horizontal Grating	Vertical Grating	Checker Board	Black	White	Horizontal Grating	Vertical Grating	Checker Board
iPhone 6	1.04	149	74	76	73	0.11	156	77	78	76
iPad 3	1.24	130	66	65	66	0.15	127	64	64	64
Laptop	2.64	124	65	64	63	0.68	125	63	62	62
4K monitor	1.50	168	82	83	81	0.16	170	81	84	82

Under all three lighting conditions, all four printed tests and all four digital displays met the criteria for both the International Council of Ophthalmology overall luminance requirement^4^ (>80 cd/m^2^) and the BS 4274–1:2003 overall luminance requirement^5^ (>120 cd/m^2^).

### Contrast

The contrast between grey background and finest grating was moderately low for TAC and KACI tests and markedly higher for the LP test (Table 4; see also Fig. 6, upper panels, represented by vertical separation of pairs of open and closed symbols). Viewing under the brighter cold or warm lighting conditions either made no difference to contrast (LP test) or modified contrast modestly (TAC and KACI test). The second finest gratings had a higher contrast relative to grey backgrounds than the finest grating for the TAC test, and the reverse was found for the LP tests and for the KACI, except under warm light, where the contrast was similar for both the finest and second finest gratings in the KACI.

**Table 4.** Weber Contrast (%) of Gratings Versus Grey Background for TAC: Teller Acuity Cards, KACI: Keeler Acuity Cards for Infants and LP: Lea Paddles

	Ambient Light (450 Lux)		Cold Light (5500K, 3682 Lux)		Warm Light (3200K, 3432 Lux)	
	Finest Grating	Second Finest Grating	Finest Grating	Second Finest Grating	Finest Grating	Second Finest Grating
TAC	0.3	−5.7	2.6	−4.5	1.8	−4.5
KACI	−1.7	0.5	−1.5	0.8	−3.4	−3.4
LP	−26	−12	−27	−12	−27	−10

For TAC, where background grey luminance varies by card (Table 2), contrast was calculated between the grating and the grey background of the same card.

### Uniformity

Printed tests showed excellent uniformity (97.4%–99.5%) under all three lighting conditions. Illuminating under studio lights marginally decreased the uniformity of three tests (KACI, LP, and CAC) and negligibly increased (warm) or decreased (cold) the uniformity of the TAC test. Variations in uniformity with lighting condition were less than 1% for all four printed tests (Table 5).

**Table 5.** Uniformity of Tests Under the Different Lighting Conditions (%)

	Ambient Light (450 Lux)	Cold Light (5500K, 3682 Lux)	Warm Light (3200K, 3432 Lux)	Lights Off
TAC	98.0	97.6	98.5	NM
KACI	99.5	99.4	99.0	NM
LP	99.4	97.8	98.9	NM
CAC	97.7	97.5	97.4	NM
iPhone 6	91.3	NM	NM	90.8
iPad 3	90.2	NM	NM	89.1
Laptop	93.5	NM	NM	88.5
4K monitor	93.1	NM	NM	94.0

NM, not measured; see Luminance.

Digital displays had poorer uniformity (88.5%–94.0%) than the printed tests, but exceeded the requirements of the BS 4274–1:2003 uniformity standard^5^ (uniformity >80%) in both lights-on and lights-off conditions. Uniformity was slightly better under ambient lighting: with the lights off, three displays had decreased uniformity, most marked (5%) for the laptop. The 4K monitor had marginally better uniformity with the lights off. Other than the laptop, variations in uniformity with lighting condition was around 1% (Table 5).

### Spectrometry

**Figure 7.**
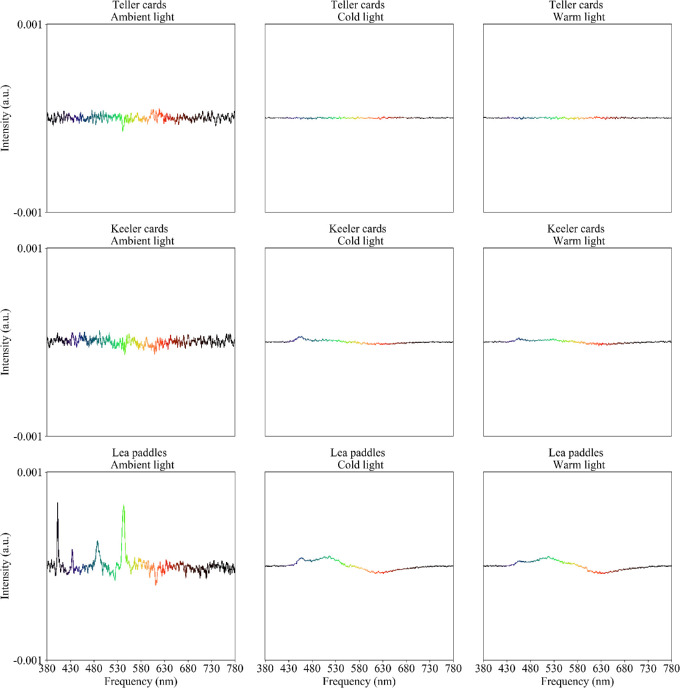
Differential spectrometry between grey background and finest grating for the printed tests. Positive values indicate the grey background has higher values at a particular wavelength than the grating. Each row represents a printed test and each column represents a lighting condition. Intensity (*vertical axis*) is expressed in the arbitrary units (a.u.) returned by the spectrometer. Ambient light level is 347 lux. The data plots are color-coded to illustrate the relevant color of the visible spectrum.

Difference spectra for three of the four printed tests (TAC, KACI, and LP) (Fig. 7) indicated a small spectral difference between grey background and finest grating for the TAC and KACI tests. The LP test show more marked differences in spectral content, possibly underpinning a difference in perceived color, especially under ambient lighting, with the grey background having relatively intense spikes around 400, 500, and 550 nm, in the blue and green regions of the spectrum, suggesting the finest gratings and grey backgrounds are not closely matched in color for the LP test. Large variability of the color spectra, akin to noise, is evident under ambient light, but not under the stronger cold and warm studio lighting.

**Figure 8.**
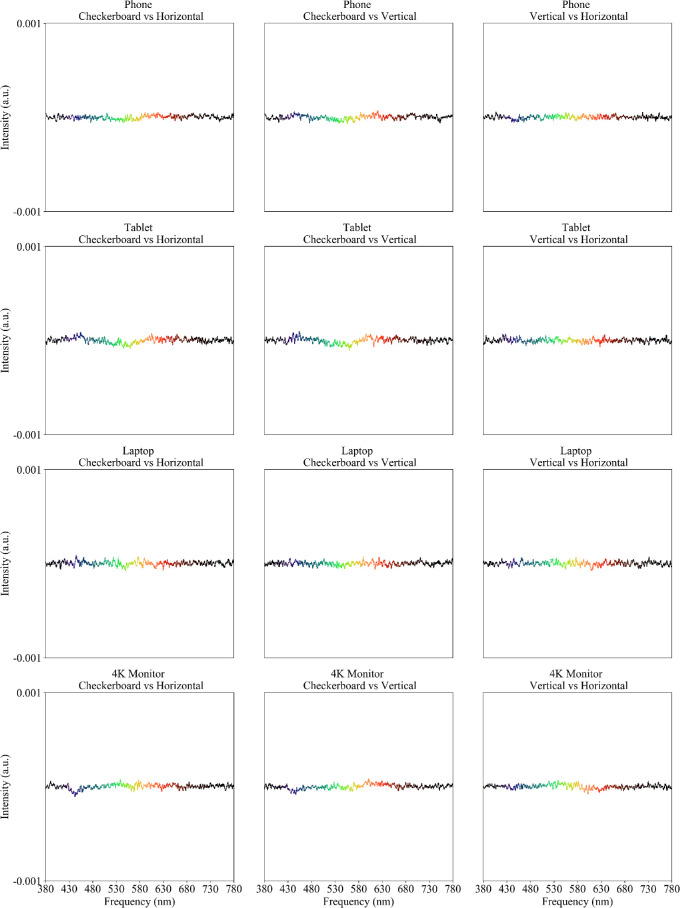
Differential spectrometry for the four digital displays by row. Each plot illustrates the difference between grey backgrounds generated by pairs of patterns (*left column*, checkerboard vs. horizontal grating; *center* *column*, checkerboard vs. vertical grating; *right column*, vertical vs. horizontal grating). Intensity (*vertical axis*) is expressed in the arbitrary units (a.u.) returned by the spectrometer. The data plots are color coded to illustrate the relevant color of the visible spectrum. See also Figure 5.

A comparison of the spectra among the three digital patterns (checkerboard, horizontal grating, and vertical grating) is shown in Figure 8. The graphs show little difference in the emitted spectra of the three patterns.

### Psychophysical Tests

**Figure 9.**
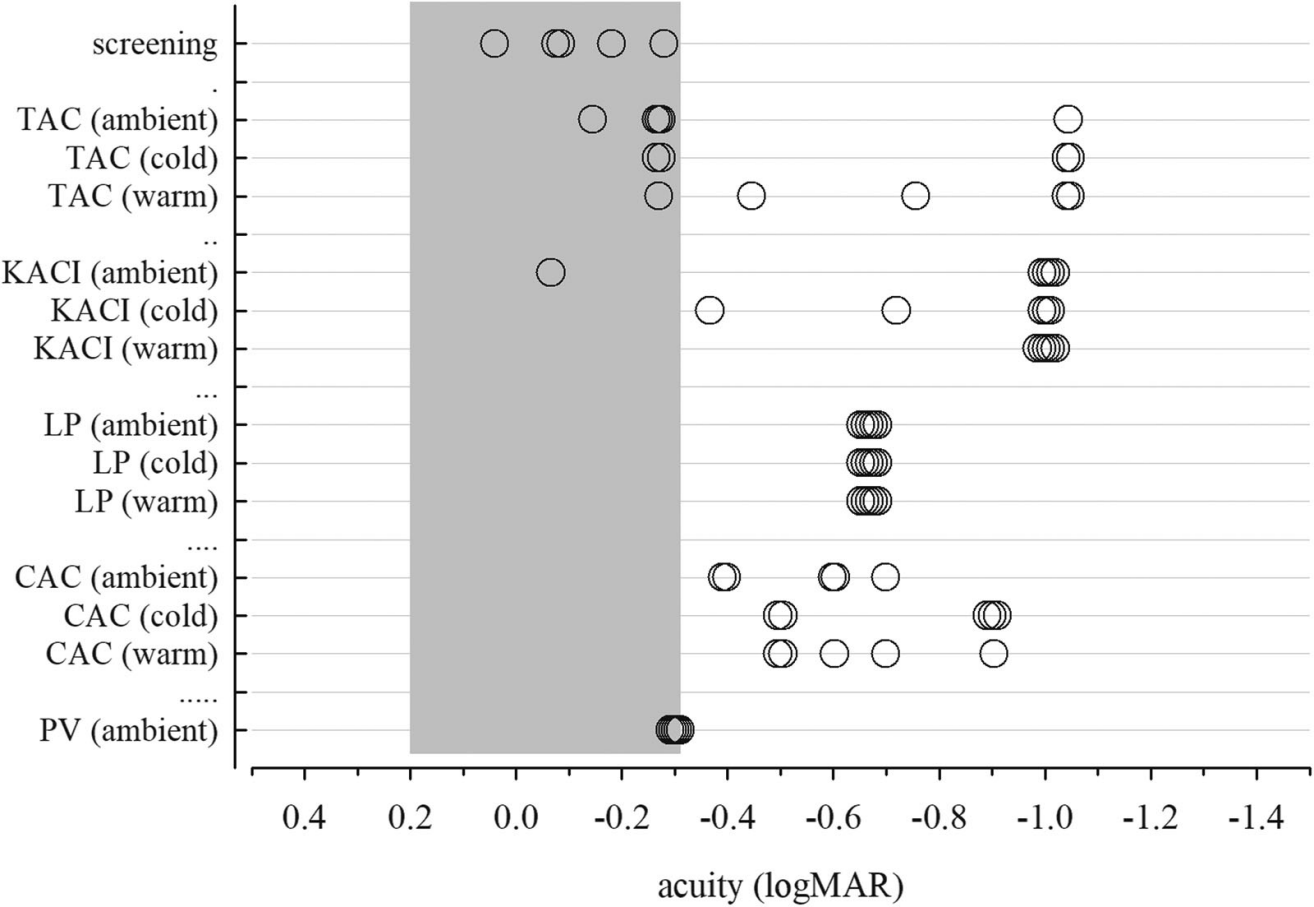
Psychophysical acuity results. Each horizontal line presents the five subjects’ results for one test and lighting condition. Identical data have been slightly splayed for better visualization. The grey zone represents normal, plausible acuities.^9^^,^^10^ Data points to the right of the grey zone represent physiologically implausible results. The screening test was crowded ETDRS, Early Treatment Diabetic Retinopathy Study. LogMAR, logarithm of the minimum angle of resolution.

For all four printed tests under all three lighting conditions, improbably good psychophysical acuities were recorded, suggesting that the small mismatches in luminance or color contrast might be at least partly responsible for making the whole target area visible, even when the constituent gratings were not theoretically resolvable (Fig. 9). Adult acuity is considered normal when it is better than 0.200 logMAR^9^ and can feasibly reach −0.200 or even −0.300 logMAR.^10^ The five subjects’ acuities all fell within this range with the screening test (crowded Early Treatment Diabetic Retinopathy Study).

The TAC test's 19 cy/cm card was the threshold card for at least one subject under all three lighting conditions, which corresponds to a physiologically unfeasible acuity of −1.044 logMAR. Although little spectral difference was found between grey background and gratings for the TAC (Fig. 7), Weber contrasts for the second finest gratings ranged from −4.5% to −5.7% depending on lighting conditions, indicating backgrounds brighter than targets (Table 4). Physiologically plausible acuities (−0.145 and −0.270 logMAR) were also recorded from some subjects.

Similarly, the KACI test's finest available grating, 17.3 cy/cm, was discernible by most subjects at 10 m under all three lighting conditions, corresponding to an improbably good acuity of −1.003 logMAR. Small spectral and luminance differences were found between grey background and gratings for the KACI (Fig. 7, Table 4), which might be at least partly responsible for the artefactually supranormal acuities. KACI targets are circled with a fine line to minimize edge effects, which may also affect visibility.

The LP test resulted in implausibly good acuity in every subject under every test condition (finest available grating of 8 cy/cm, equivalent to −0.668 logMAR at 10 m). LPs had the greatest spectral difference (Fig. 7) and Weber contrasts (−26% or −27%, indicating markedly brighter backgrounds, Table 4) between grey background and finest grating target.

The CAC also resulted in implausibly good acuity in every subject and under every test condition (−0.398 to −0.903 logMAR). Neither luminance nor spectroscopic measures of the CAC finest detail (three lines rather than an extended grating) were possible, and so any eventual photometric issues underlying these implausibly good acuities are not clear.

The PV test level “6/60 at 50 cm” was the threshold level at 10 m for all subjects, in keeping with borderline plausible acuity of −0.301 logMAR. There was a surprising lack of variability across subjects. Since the black and white gratings for PV are comprised of the same pixels as the background grating, neither luminance nor color contrast are likely to affect this acuity measure.

## Discussion

Black grating luminance varied most across the printed tests, whereas white gratings varied least, consistent for all lighting conditions. Although this may not represent any problem in itself, it reinforces the idea that there is a lack of standardization and regulation of infant acuity tests. The luminance of grey backgrounds and luminance of finest or second -finest gratings did not always match closely, and the mismatch was most pronounced for the LP test, with a notably brighter background producing a Weber contrast of 27% with the finest grating target. Lighting condition affected luminance as expected, because the ambient light was relatively dim but had little effect on contrast. For the digital displays, room lighting on or off had little effect on luminance, and grey backgrounds had very similar luminance whether generated by a checkerboard or by vertical or horizontal gratings. Although no national or international standards for luminance and luminance uniformity exist for acuity tests for infants and young children, under all three lighting conditions, all four printed tests and all four digital displays met luminance and uniformity standards developed for adult tests.^4^^,^^5^

The manufacturers of the TAC note that the different luminances measured in the grey background of different cards is part of their manufacturing quality control process: in said process, each card's grating and background are matched perceptually by an adult with normal vision.^8^ This matching seems to be relatively effective depending on the card (according to our measurements, the finest grating card has a better match than the second finest grating). In contrast, the KACI seem to have a more consistent grating and background luminance across different cards. However, the process by which KACI are manufactured is unknown, so it is not possible to know if they follow the same method of perceptual match or another one. This factor brings up two points of discussion: Is photometric match irrelevant to these tests if there is a perceptual match? Is a perceptual match for one person the same for all people? These discussion points, while interesting, are out of the scope of this article, which only aims to assess photometric qualities, and would require specific studies to be answered.

A perceptual brightness match between the gratings and grey background is a fundamental requirement of these tests. Any perceptible difference between grating and background might trigger a response, not because the spatial frequency of the grating had been resolved, but because the much larger area of luminance mismatch had been resolved. This factor could lead to an incorrect, excessively good acuity and potentially to false-negative results in vision testing in young populations (e.g., amblyopia screening). We consider luminance match to be a key area for compliance of acuity tests based on a preferential looking task; however, no standards exist.

The difference in spectrometry was used to measure potential differences in color between the different measured targets. In the graphs shown, a flat line represents that there was little to no difference in color spectra, indicating no difference in color. In contrast, nonflat graphs represent a difference in color spectra, which suggests there may be a difference in color between the two targets. As such, a peak in the graphs indicates color dominance of one target over the other in that specific region of the color spectrum. Spectrometry of the printed tests showed greater variability, possibly noise, under ambient light than under the cold or warm studio lights; we have no explanation for this phenomenon. Both TAC and KACI tests had rather flat difference spectra (between grey background and finest grating), suggesting relatively little color dominance. The LP test showed higher spectral disparity under ambient light conditions, which was less under studio light illumination. The large difference in the blue and green regions of the spectrum, creating a color dominant, could be due to the flat grey paint used for the background of the LP test. The alternative use of a pattern of fine black dots over a white background to create the grey, presumably based on the same black and white used to create grating targets, explains the much better spectral compliance of the other printed tests (Fig. 4). Greys generated by fine patterns on the digital displays did not have major spectral differences which might have been expected owing to “pixel bleeding,” where white pixels bleed color to the adjacent black pixels (Fig. 5).

These findings suggest potential advantages of digital displays over printed tests, because they are minimally affected by the light conditions used here, and can be calibrated to match other devices or to conform to new standards. They are, however, reliant on the user to conform to photometric standardization procedures, for example, having auto-brightness off or on. They are not vulnerable to the same luminance contrast difficulties encountered with printed tests, or to fading or dirtying over time. However, it is not clear whether the luminance and spectral contrasts described here, especially for the LP test, have clinically meaningful consequences.

Our preliminary psychophysical test results suggest that, for young, normally sighted adults, the luminance contrasts and/or spectral differences measured in three of the four printed tests (it was not possible to measure the CAC test) can produce artificially good visual acuities, surpassing even the extraordinary acuity of the peregrine falcon.^11^ While edge effects that incorrectly increase the measured acuity have been observed in the TAC test,^12^ our apparent high acuities are as much as an order of magnitude higher than these edge effects.

A floor effect is suspected for the LP test for two reasons: first, all participants were able to successfully identify the finest grating available under all illuminating conditions. Second, its finest grating was relatively coarse compared to the other tests: 8.0 cy/cm from LP compared with the KACI, which reached 17.3 cy/cm and the TAC which reached 38.0 cy/cm. If a floor effect was indeed present for the LP test, it could also explain the lack of variability.

Distances greater than 10 m were not tested in the present study because a comprehensive psychophysical experiment was not the objective of this study, but would be interesting to explore in future work.

The PV test performed on an iPad 3 produced acuities at the upper limit of plausible values in the same adults, with an unexplained lack of variability. Further studies with larger numbers are desirable to explore these findings.

Even if the results represent a real finding for young adults, their clinical relevance may be less in infants and young children, the target patient group for these tests, in whom acuity, contrast sensitivity, and color sensitivity remain immature.^13^^,^^14^

Testing acuity at 10 m markedly deviates from manufacturers’ instructions, which recommend test distances between 25 cm and 1 m. At this closer range, grating patterns or optotypes may stimulate a relatively extrafoveal, that is, lower resolution retinal locus which, in turn, may fail to induce a foveating saccade, that is, looking response, in a child. In contrast, the 10 m distance used in this study ensured all targets stimulated the adults’ high-resolution fovea.

There are several aspects of this study that could be strengthened in future research. The photometer was used at a close distance with a close-up lens, because this strategy allowed for the best control of the illumination conditions over the cards and electronic devices. The magnifying effect of the lens decreased the number of grating cycles in the measuring area, potentially including partial cycles and thus increasing variability. During photometric measurements, it was not possible to fully standardize the light illuminating the test cards or displays; even the position of the researcher could affect the illuminance. We cannot exclude the possibility that paper or plastic surfaces exhibited fluorescence excitable in the wavelength range of light sources used; if so, findings may not be generalizable to sunlight or incandescent lighting with significantly different ultraviolet contents. The printed tests used were in active service across various hospitals and academic units and, although generally in good condition, showed signs of wear and tear, and even may have included cards of different and unknown ages, all of which might increase variability owing to dirtiness, fading, or different print runs. Furthermore, three subjects spontaneously commented that the angle at which the printed tests were held during psychophysical testing affected the target visibility. A fixed, wall-mounted option might be preferable; however, holding the tests by hand emulates the usual clinical setting.

The PV test was not assessed under cold and warm light conditions because it is an emissive display. Indeed, emissive display immunity to differing lighting conditions has not been explicitly demonstrated in this study as covered by the related photometric characterization standards.^15^

Clinical infant vision tests have not changed for decades,^16^^,^^17^ using a printed surface with the ageing and fading properties of paper and ink. The evolution of digital display technologies and their use for vision testing^1^^,^^2^^,^^6^^,^^7^^,^^18^^–^^20^ is likely to continue and to require regulatory compliance to include photometric standards.

## Conclusions

To the best of our knowledge, photometric evaluations of printed infant acuity tests have not been published. No standards for the physical or photometric properties of infant printed tests exist. Given that the central premise of preferential looking testing assumes equal background and target luminance, such a standard seems reasonable. The findings from the current study suggest acuity tests routinely used for clinical testing of infants and young children have mismatches of luminance and spectra which are perceptible to healthy young adults, creating artificially good acuity measurements.
